# Knockout of the ATPase inhibitory factor 1 protects the heart from pressure overload-induced cardiac hypertrophy

**DOI:** 10.1038/s41598-017-11251-8

**Published:** 2017-09-05

**Authors:** Kevin Yang, Qinqiang Long, Kamalamma Saja, Fengyuan Huang, Steven M. Pogwizd, Lufang Zhou, Masasuke Yoshida, Qinglin Yang

**Affiliations:** 1 Department of Nutrition Sciences, University of Alabama at Birmingham, Birmingham, Alabama 35294 USA; 2 Departments of Internal Medicine and Institute of Hypertension, Tongji Hospital, Tongji Medical College, Huazhong University of Science and Technology, Wuhan, P. R. China; 3 Department of Biochemistry, University of Kerala, Thiruvananthapuram, Kerala 695 581 India; 4 Division of Cardiovascular Diseases, Department of Medicine, University of Alabama at Birmingham, Birmingham, Alabama 35294 USA; 5 Department of Molecular Bioscience, Kyoto Sangyo University, Kamigamo-Motoyama, Kyoto, 603-8555 Japan

## Abstract

Mitochondrial ATP synthase catalyzes the coupling of oxidative phosphorylation. Under pathological conditions, ATP synthase hydrolyzes ATP to replenish protons from the matrix into the intermembrane space, sustaining mitochondrial membrane potential. ATPase inhibitory factor 1 (IF1) is a nuclear-encoded, ATP synthase-interacting protein that selectively inhibits the hydrolysis activity of ATP synthase, which may render the protective role of IF1 in ischemic hearts. However, the *in vivo* cardiac function of IF1 and the potential therapeutic application targeting IF1 remain obscure. In the present study, we uncovered that IF1 is upregulated in mouse hearts with pressure overload-induced hypertrophy and in human hearts with dilated cardiomyopathy. IF1 knockout (KO) mice were protected against cardiac dysfunction and pathological development induced by transverse aortic constriction (TAC) or isoproterenol infusion. The reduced ATP hydrolysis activated AMPK activity in IF1 KO hearts, which together facilitated autophagy. These results suggest that IF1 upregulation in the failing heart may be a maladaptive response. Inhibiting IF1 in the hypertrophied heart not only prevents cell death from excessive mitochondrial depolarization but also activates AMPK signaling and increases autophagy. Therefore, IF1 inhibition may serve as a potential therapeutic target in treating pathological cardiac hypertrophy and heart failure.

## Introduction

Prolonged cardiac hypertrophy in response to pathological stresses is the main risk factor for congestive heart failure, a leading cause of mortality and morbidity^1^. Despite substantial advances in therapy, successful treatment of pathological hypertrophy and heart failure remains challenging.

Mitochondria play a pivotal role in cellular energy status and cell fate. ATP synthase is a crucial protein complex in the mitochondria that couples osmo-chemical energy into ATP. ATP synthase not only regulates the production and hydrolysis of ATP in mitochondria but also is a key regulatory mechanism of mitochondrial membrane potential (ΔΨm)^2, 3^. ATP synthase consists of two sub-complexes with distinct but complementary functions. The Fo complex contains transmembrane subunits that transport protons, while the F1 is a peripheral complex on the matrix side that contains the catalytic nucleotide binding sites for ATP synthesis^4–7^. Fo and F1 are coupled through two stalk-like subunit connections: a central rotor shaft and a peripheral stator^4^. Despite the well-recognized role of the ATP synthase in energy metabolism, the effect of changes in ATP synthase activities on cardiac pathogenesis remains obscure.

The inhibitory factor 1 (IF1) is a nuclear-encoded ATP synthase interacting protein that suppresses the hydrolysis activity of ATP synthase^8, 9^ under acidic conditions, such as in myocardial ischemia^10–12^. The roles of IF1 in various tissues remain controversial. It has been shown that IF1 mimetic compounds improve cardiac function in isolated heart subjected to ischemia-reperfusion^13, 14^. Transgenic overexpression of a mutant IF1 protects neuronal damages^15^. IF1 deficiency has been linked to anemia due to impaired heme synthesis^16^. In contrast, loss of IF1 in mice strongly protects against antimycin-induced electron transport chain dysfunction and cell death^17^. The apparently contradicting effects of IF1 in various tissues are puzzling and further studies are warranted. While IF1 knockout in mice leads to no overt phenotype^18^, it remains unclear if IF1 in the *in vivo* heart becomes crucial in maintaining cardiac function under pathological stresses.

In the present study, we hypothesize that cardiac IF1 regulates mitochondrial polarization, energetic function and cell fate in pathological hypertrophy as an ATPase inhibitor of ATP synthase. To test this hypothesis, we investigate how the loss of cardiac IF1 affects cardiac structure/function in response to pathological stimuli and define the underlying molecular and biochemical mechanisms.

## Results

### IF1 protein expression is upregulated in pressure overload-induced hypertrophied hearts

**Figure 1:**
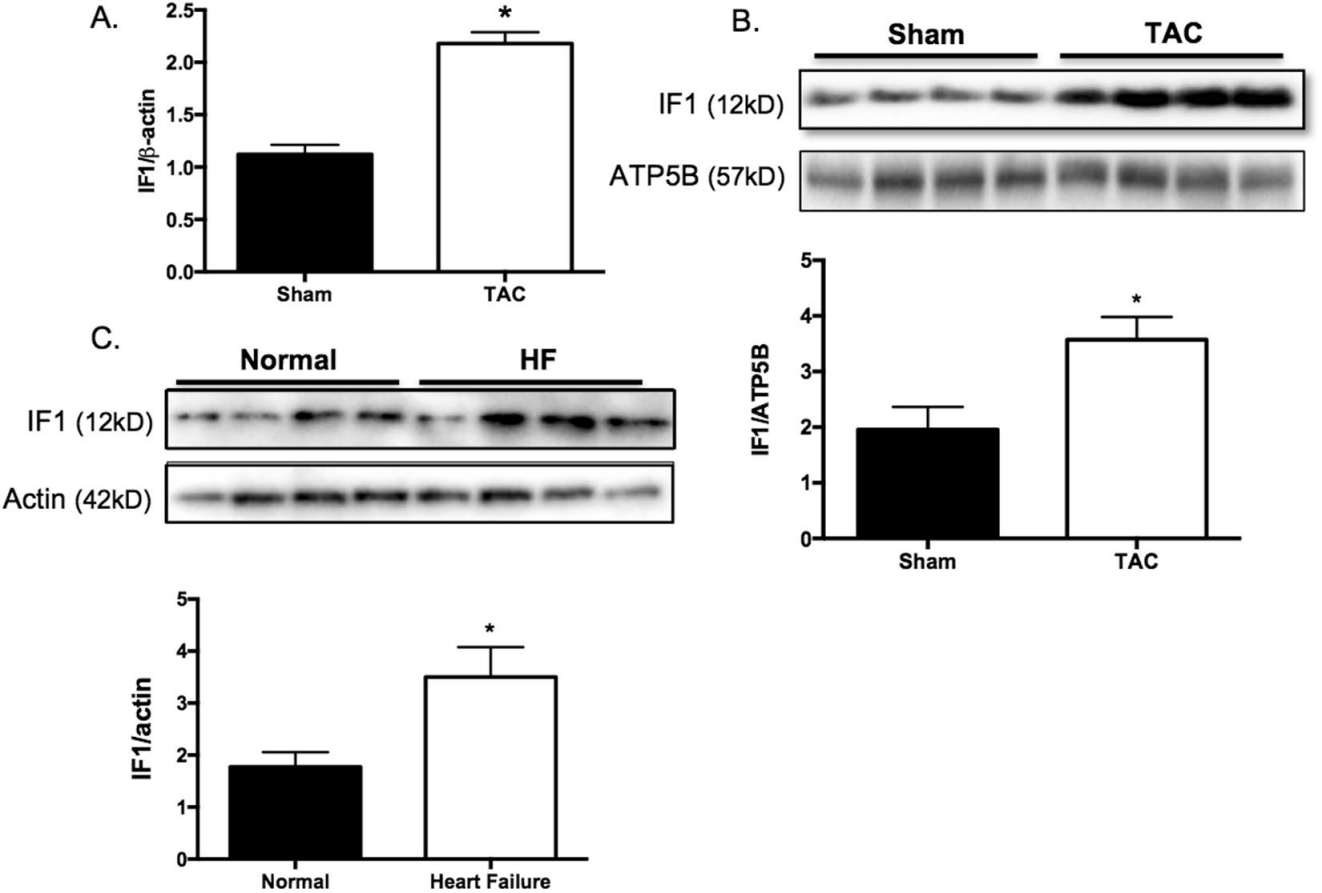
IF1 expression in the pathological heart. (**A**) Transcript expression of IF1 was determined by real-time PCR. Transcript level of β-actin was used as loading control. Mice were subjected to 4 weeks of TAC. RNA was extracted from LV tissue (n = 4). (**B**) Protein expression of IF1 and ATP5B were determined by Western blot. Actin was used as protein loading control. The ratio of IF1/ATP5B is shown. Mice were subjected to 4 weeks of TAC. Protein was extracted from LV tissue (n = 4). (**C)** Protein expression of IF1 was determined by Western blot. Actin was used as protein loading control. Protein lysates were obtained from heart samples from heart failure patients and healthy donors (n = 4). *P < 0.05 vs sham. Data are expressed as mean ± SEM.

We first investigated cardiac IF1 expression in the normal and hypertrophic hearts. Real-time PCR revealed that IF1 mRNA was upregulated in the heart in response to TAC-induced pressure overload (Fig. 1A). Western blot analysis demonstrated IF1 protein levels i﻿ncreased almost 2-fold in hypertrophied compared with the sham control hearts (Fig. 1B). Western blot analysis on protein lysates from patients with dilated cardiomyopathy and healthy donors revealed that cardiac IF1 protein was upregulated by about 55% in human failing hearts compared to nonfailing donor hearts by about 55% (Fig. 1C). These results provide the first evidence that cardiac IF1 is upregulated in pathologic hearts.

### IF1 deficiency protects the heart against pressure overload hypertrophy

**Figure 2:**
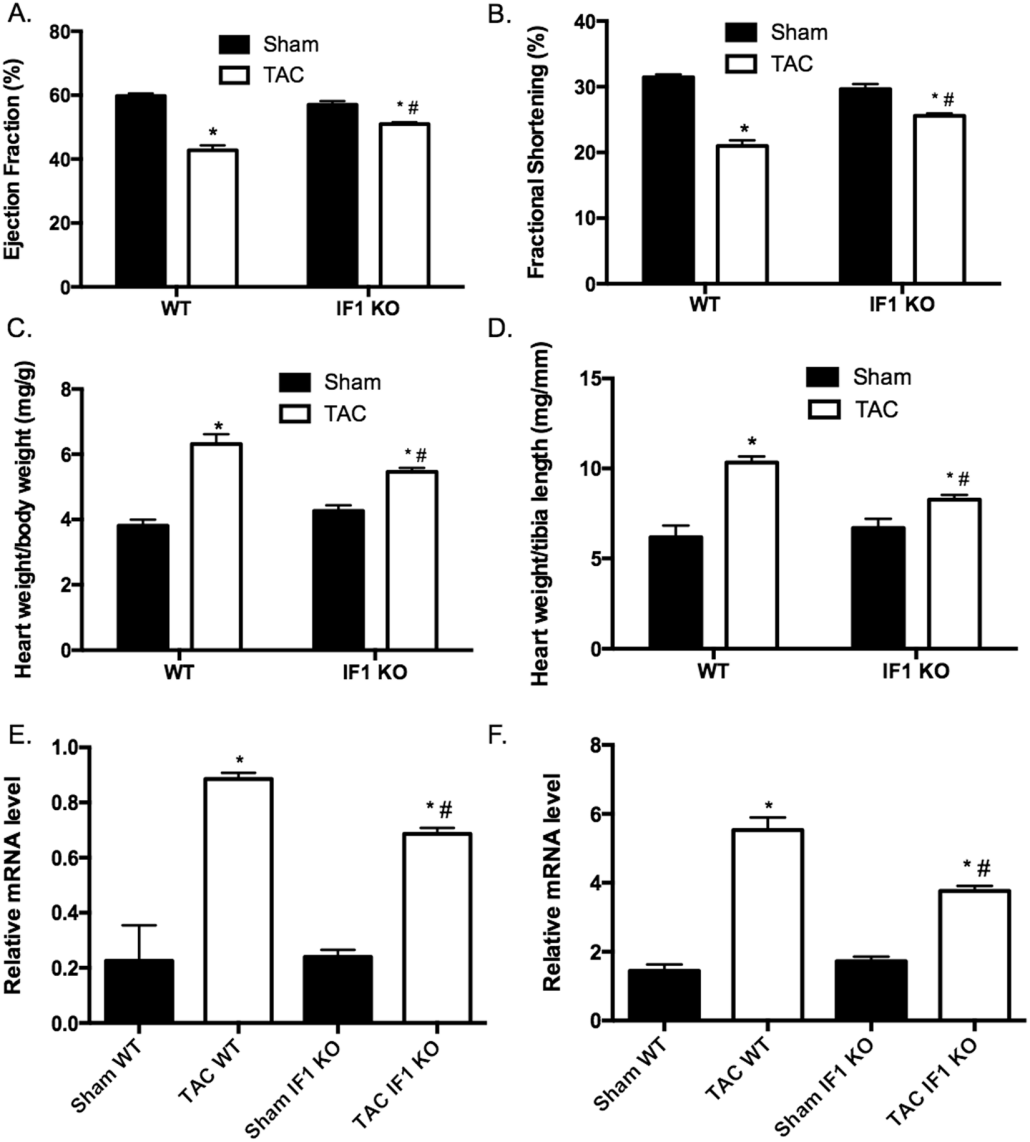
Echocardiographic parameters in mice with pressure overload. (**A)** and (**B)** echocardiographic results of ejection fraction (%), and fractional shortening (%) are shown 4 weeks after TAC (n = 5–9). (**C)** Ratio of heart weight (mg) to body weight (g). (**D**) Ratio of heart weight (mg) to tibia length (mm). (**E**) and (**F)** Real-time PCR assessment of natriuretic peptide A and B transcript expression normalized to 36B4. RNA was extracted from LV tissue of hearts subjected to 4 weeks of TAC (n = 4). *P < 0.05 vs sham, ^#^P < 0.05 vs WT TAC. Data are expressed as mean ± SEM.

**Figure 3:**
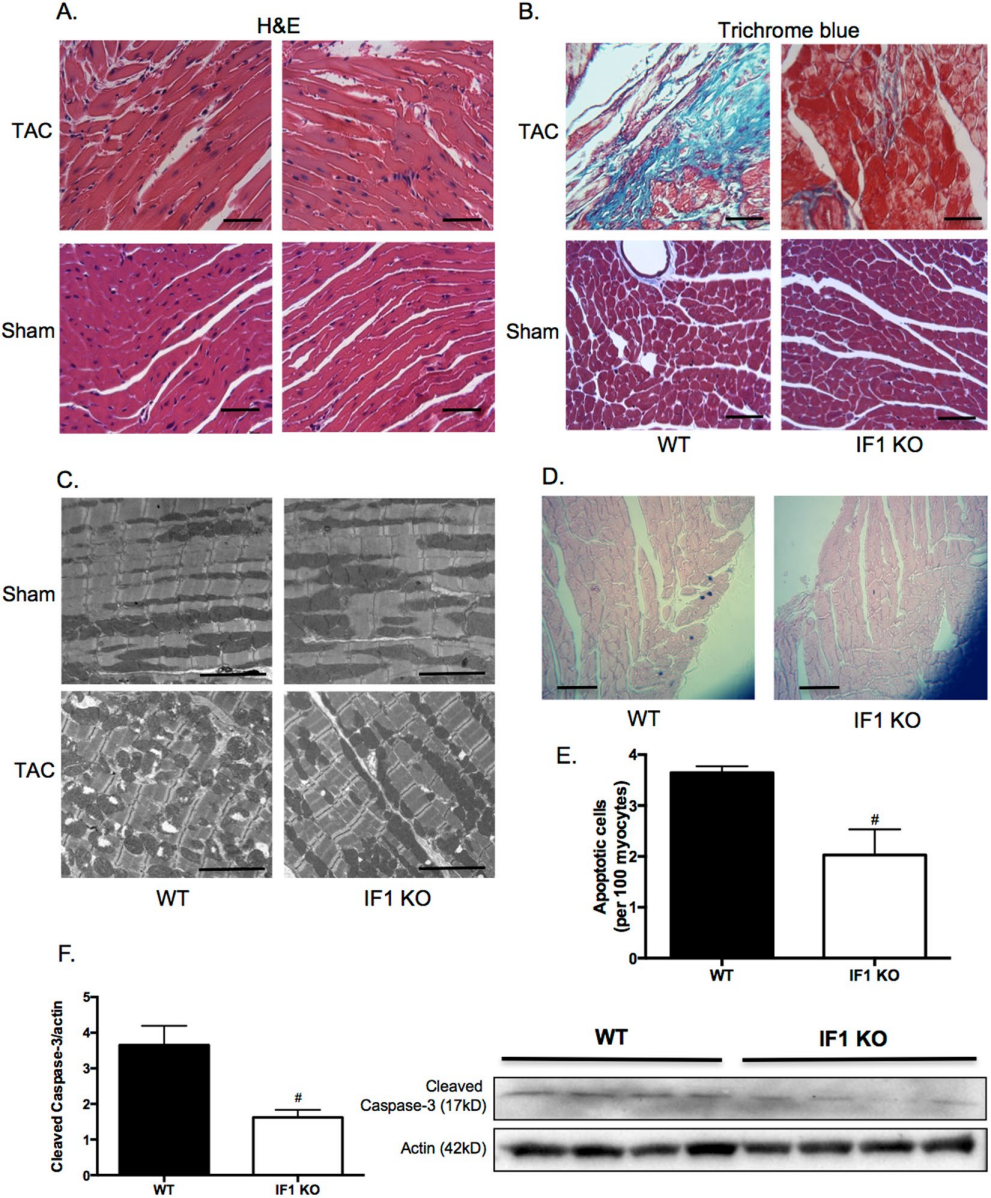
Cardiac histology and ultrastructure and apoptosis assessment in mice with pressure overload. Mice were subjected to TAC for 4 weeks. (**A**) Representative histological images (x400) with H&E staining on heart sections of IF1 KO and WT hearts subjected to TAC and Sham operation. Scale bar: 50 μm. (**B**) Representative images (x400) of heart sections stained with Trichrome blue of IF1 KO and WT hearts subjected to TAC and Sham operation. Scale bar: 50 μm. (**C**) Representative images of LV transmission electron microscope (TEM) assessment (x1100). Scale bar: 5 μm. (**D**) Representative image of apoptotic cells in a heart section assessed by TUNEL assay. Scale bar: 50 μm. (**E**) Quantification results of TUNEL assay (n = 4). (**F**) Western Blot results of caspase-3 (n = 4). ^#^P < 0.05 vs WT TAC. Data are expressed as mean ± SEM.

We further confirmed the previous finding^18^ that no overt phenotype was present in IF1 KO mice up to 8 months of age. IF1 KO mice (10–12-week-old) were subjected to TAC-induced pressure overload for 4 weeks. Echocardiographic assessment confirmed a sustained LV pressure overload with an average intraventricular pressure gradient of ~40 mmHg (Table 1). Furthermore, cardiac contraction, as indicated by fractional shortening and ejection fraction, was improved in IF1 KO relative to the wild type hearts (Fig. 2A,B). TAC-pressure overload induced a substantial increase of inter-ventricular septum (IVS) and posterior wall thickness (LVPW) at both systole and diastole in WT, but not in IF1 KO mice (Table 1). Interventricular dimensions (LVID) at systole and diastole were increased in WT, but not in IF1 KO mice (Table 1). Heart weight to body weight and heart weight to tibia length ratios were lower in the IF1 KO than in the WT hearts (Fig. 2C,D). Furthermore, Real-timePCR revealed that cardiac expression of molecular markers of cardiac hypertrophy, such as natriuretic peptide A (*Nppa*) and B (*Nppb*), was less pronounced in IF1 KO than in WT mice (Fig. 2E,F). Histological examination with H/E and Trichrome Blue staining on heart sections demonstrated smaller cross-sectional area of cardiomyocytes (Fig. 3A) and less pronounced fibrosis (Fig. 3B) in IF1 KO than WT mice after TAC. Transmitted electron microscopy (TEM) imaging of heart sections showed normal mitochondrial and sarcomeric integrity in cardiomyocyte ultrastructure between the IF1 KO and WT control hearts (Fig. 3C). After TAC, WT hearts showed mitochondrial network disruption with loss of matrix compared with IF1 KO hearts (Fig. 3C). TUNEL assay detected fewer apoptotic cardiomyocytes on heart sections from IF1 KO than WT hearts after TAC (Fig. 3D,E). Consistently, Western blot showed reduced cleavage (17 KD) of caspase-3 on the protein extracts from post-TAC IF1 KO compared to post-TAC WT hearts (Fig. 3F).

**Table 1:** Echocardiography measurement in mice with pressure overload.

Parameters	WT		IF1 KO	
	Sham (n = 5)	TAC (n = 7)	Sham (n = 6)	TAC (n = 9)
IVSd, mm	0.67 ± 0.06	0.92 ± 0.10^*^	0.69 ± 0.08	0.80 ± 0.11
IVSs, mm	0.97 ± 0.04	1.38 ± 0.11^*^	1.13 ± 0.07	1.16 ± 0.15
LVIDd, mm	4.19 ± 0.10	4.48 ± 0.16	4.17 ± 0.11	4.04 ± 0.07^†^
LVIDs, mm	2.90 ± 0.08	3.52 ± 0.14^*^	2.96 ± 0.07	2.97 ± 0.06^†^
LVPWd, mm	0.46 ± 0.04	0.87 ± 0.09^*^	0.51 ± 0.03	0.73 ± 0.06^†^
LVPWs, mm	0.69 ± 0.06	1.01 ± 0.10^*^	0.80 ± 0.04	0.92 ± 0.05
LV mass, mg	42.40 ± 5.60	92.36 ± 12.68^*^	44.75 ± 3.25	60.30 ± 3.42^†^
Pressure Gradient, mmHg	NA	40.25 ± 3.37	NA	46.99 ± 1.09

Abbreviations: IVS;s and IVS;d: interventricular septal wall thickness (systole and diastole); LVIS;s and LVID;d: left ventricular dimension at systole and diastole; LVPW;s and LVPW;d: posterior wall thickness at systole and diastole; *P < 0.05 vs sham, ^†^P < 0.05 vs WT TAC. Data are expressed as mean ± SEM.

**Figure 4:**
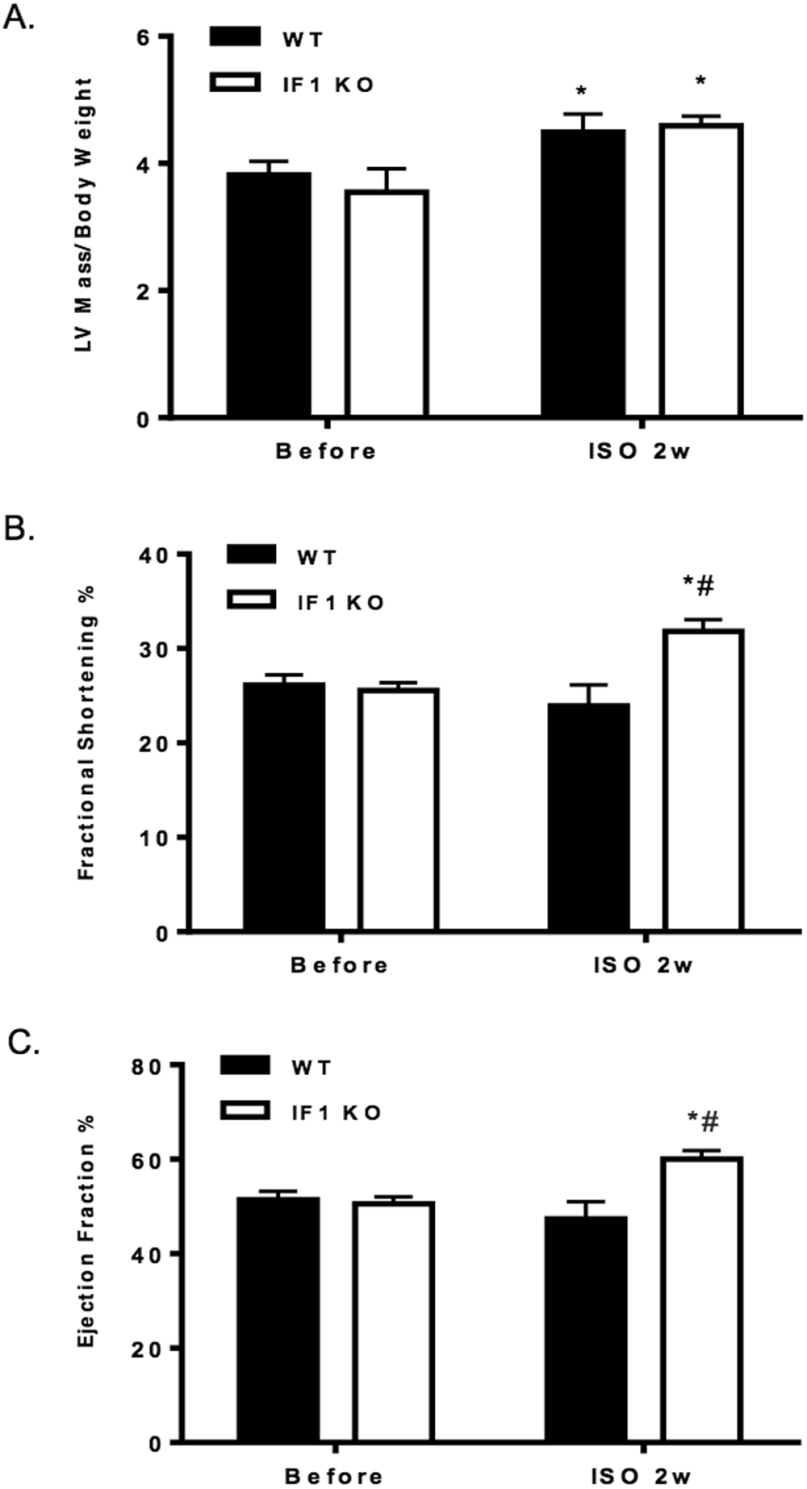
Echocardiographic parameters of mice after 2 weeks of isoproterenol infusion. (**A**) Ratio of LV mass (mg) to body weight (g) (n = 7). (**B)** and (**C**) Echocardiographic results of fractional shortening (%) and ejection fraction (%) are shown (n = 7). *P < 0.05 vs Before Isoproterenol, ^#^P < 0.05 vs WT ISO. Data are expressed as mean ± SEM.

We further investigated how IF1 KO responds to activation of adrenergic stimulation by treating the mice with isoproterenol infusion. Echocardiography showed that isoproterenol infusion increased the LV mass to body weight ratio in both IF1 KO and WT mice (Fig. 4A). IF1 KO hearts showed an enhanced contraction with greater fractional shortening and ejection fraction in response to isoproterenol compared with WT hearts (Fig. 4B,C). Moreover, WT but not IF1 KO hearts were dilated with increased LV internal dimension (LVID) at both systole and diastole (Table 2). Vehicle (PBS) treatment in mice displayed no change between WT control and IF1 KO hearts (data not shown). These results indicate that the adrenergic response in IF1 KO hearts is enhanced but hearts were protected from LV dilatation.

**Table 2:** Echocardiography measurement in mice with isoproterenol infusion.

Parameters	Before Isoproterenol		After Isoproterenol (2 weeks)	
	WT (n = 7)	IF1 KO (n = 7)	WT (n = 7)	IF1 KO (n = 7)
LVID;s (mm)	3.22 ± 0.08	3.20 ± 0.11	3.60 ± 0.11*	2.96 ± 0.11^†^
LVID;d (mm)	4.31 ± 0.09	4.29 ± 0.10	4.76 ± 0.11*	4.33 ± 0.10^†^
IVS;s (mm)	1.13 ± 0.04	1.08 ± 0.03	1.25 ± 0.04	1.36 ± 0.03
IVS;d (mm)	1.03 ± 0.06	0.96 ± 0.04	1.09 ± 0.04	1.23 ± 0.03
LVPW;s (mm)	0.88 ± 0.05	0.92 ± 0.07	0.85 ± 0.04	1.07 ± 0.07^†^
LVPW;d (mm)	0.63 ± 0.06	0.71 ± 0.09	0.57 ± 0.03	0.77 ± 0.05

Abbreviations: IVS;s and IVS;d: interventricular septal wall thickness (systole and diastole); LVIS;s and LVID;d: left ventricular dimension at systole and diastole; LVPW;s and LVPW;d: posterior wall thickness at systole and diastole; *P < 0.05 vs Before Isoproterenol, ^†^P < 0.05 vs WT ISO. Data are expressed as mean ± SEM.

### IF1 KO protects the heart from mitochondrial depolarization

**Figure 5:**
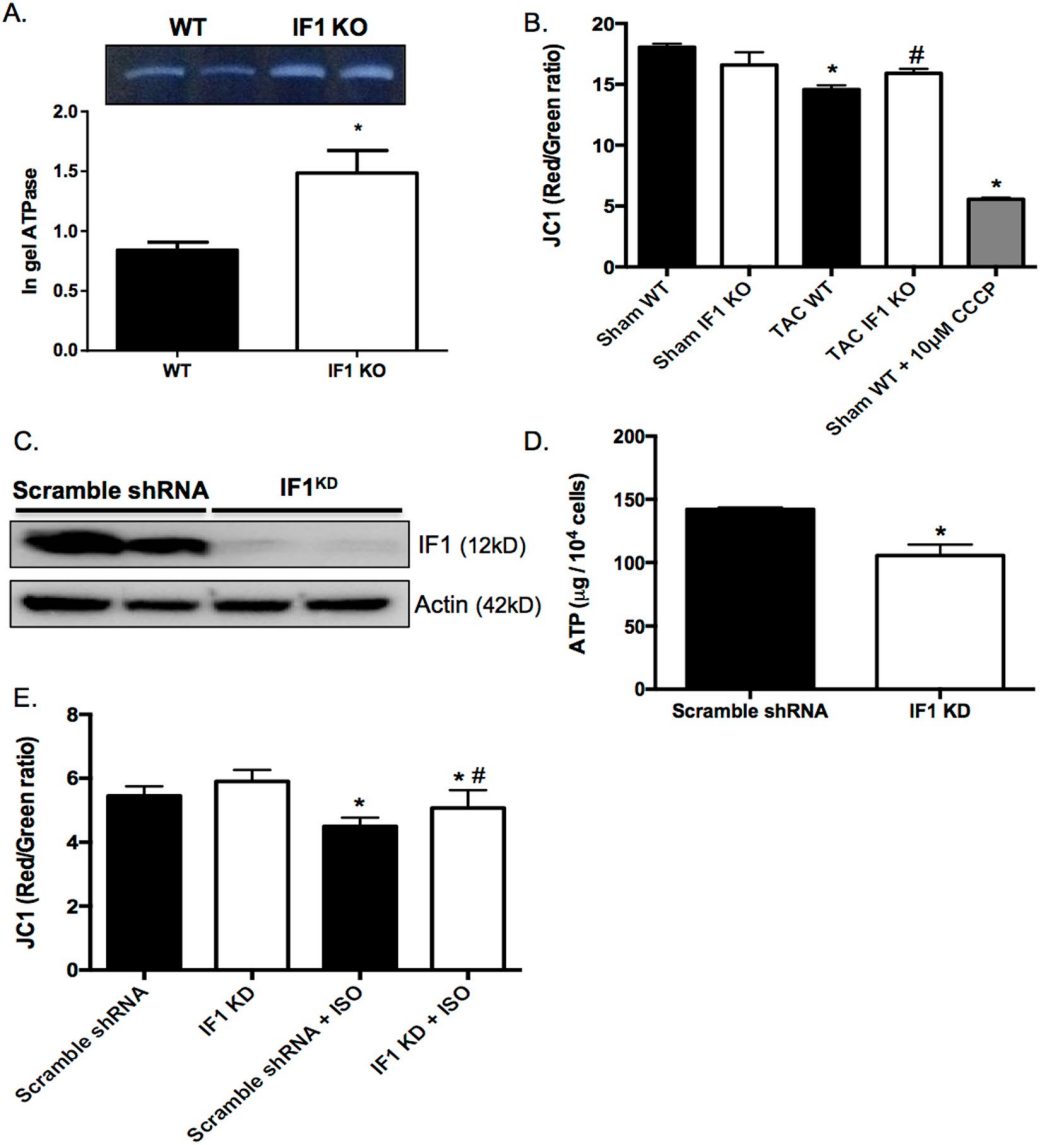
Effects of IF1 deficiency on ATP synthase activity and mitochondrial membrane potential. (**A**) In gel ATPase assay on LV mitochondria isolated from mice subjected to 4-week pressure overload (n = 4). (**B**) JC1 assay for mitochondrial membrane potential (ΔΨm) on LV mitochondria isolated from mice subjected to 4-week pressure overload (n = 6). (**C**) Western blot analysis of IF1 on protein samples from stable IF1 KD and control H9C2 cells. Protein loading was normalized to actin. (**D**) ATP content in stable IF1 KD and control H9C2 cells (n = 5). (**E**) JC-1 assay for ΔΨm on H9C2 cells treated with isoproterenol (n = 5). *P < 0.05 vs sham, ^#^P < 0.05 vs WT TAC. Data are expressed as mean ± SEM.

After confirming the complete IF1 KO in the heart, we conducted in-gel ATPase staining on mitochondrial extracts confirming an increase of ATPase activity in IF1 KO hearts (Fig. 5A). Quantitative JC-1 assay on mitochondria isolated from mouse hearts showed no ΔΨm change in IF1 KO compared with WT hearts subjected to sham procedure (Fig. 5B), whereas ΔΨm loss in IF1 KO was less pronounced than that in WT mitochondria from mice subjected to TAC (Fig. 5B). In a stable IF1 knockdown (KD) H9C2 cell line (Fig. 5C), ATP assay revealed a substantial reduction of ATP content in IF1 KD H9C2 cells (Fig. 5D). JC-1 assay consistently showed IF1 KD H9C2 cells had a less pronounced isoproterenol (1 μmol/L for 24 hours)-induced ΔΨm reduction than scramble shRNA H9C2 cells (Fig. 5E). These results indicate that pathological stress-induced mitochondrial depolarization is relatively sustained in IF1 deficient hearts.

### Loss of IF1 activates the AMPK pathway and induces autophagy in the heart

**Figure 6:**
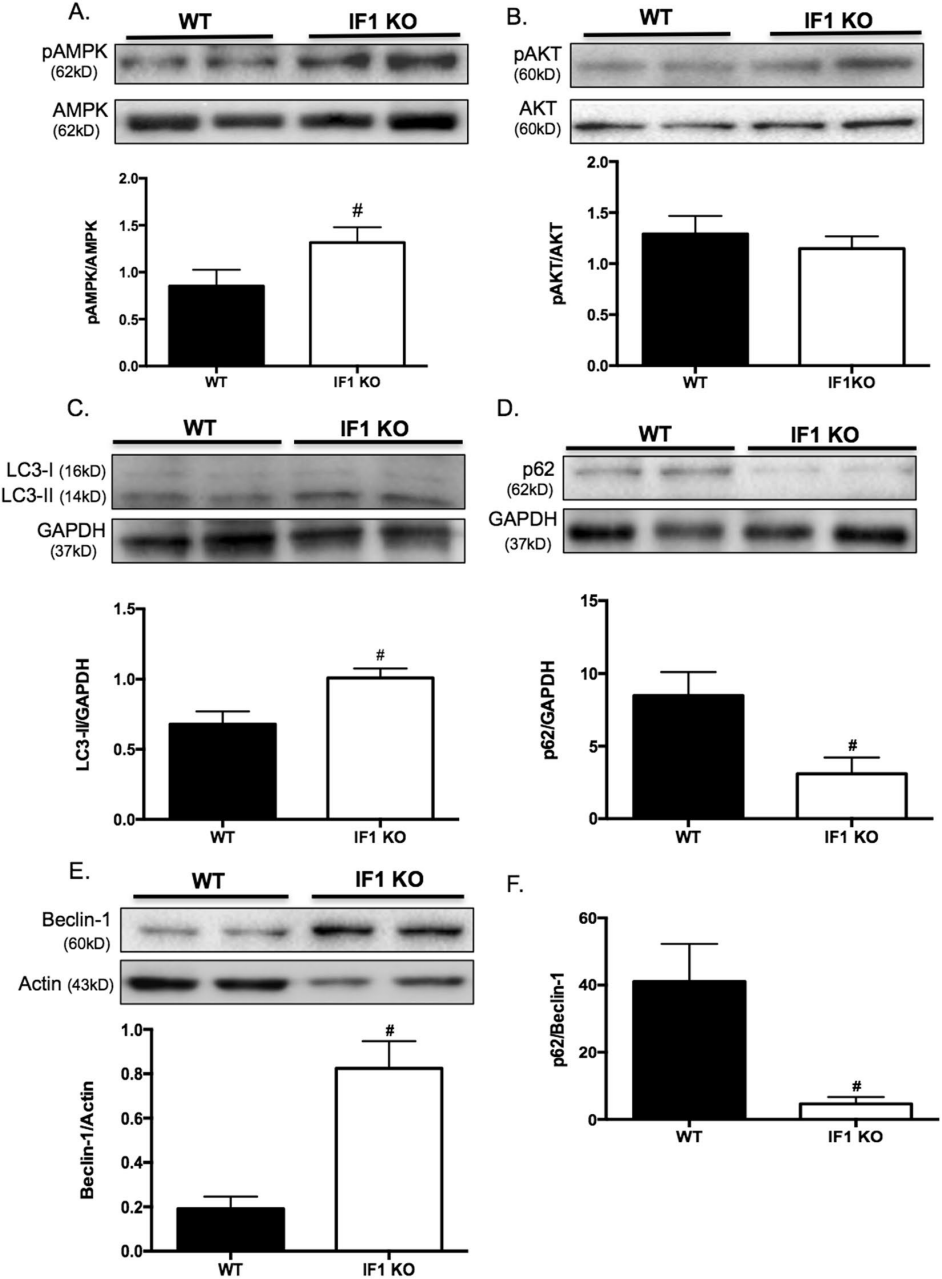
AMPK activity and autophagy in mice with pressure overload. Western blot was conducted on protein samples extracted from LV tissue of hearts subjected to TAC for 4 weeks. (**A**) Protein expression of pAMPK and AMPK. pAMPK to AMPK ratio is shown (n = 4). (**B**) Protein expression of pAKT and AKT. pAKT to AKT ratio is shown. (**C)** Protein expression of LC3. LC3-II protein levels were normalized to GAPDH (n = 4). (**D**) Protein expression of p62. Expression was normalized to GAPDH (n = 4). (**E**) Protein expression of Beclin-1. Expression was normalized to Actin (n = 4). (**F**) Ratio of p62 to Beclin-1 protein expression. ^#^P < 0.05 vs WT TAC. Data are expressed as mean ± SEM.

Western blot analyses showed that the phosphorylation of AMPK was increased on protein samples from IF1 KO compared to WT hearts after TAC, while the total AMPK protein level remained the same (Fig. 6A). The resulting increase in pAMPK/AMPK ratio (Fig. 6A) indicates the activation of AMPK in IF1 KO hearts. On the other hand, the phosphorylation of AKT showed no difference between IF1 KO and WT mice subjected to TAC (Fig. 6B). Further, Western blot analyses revealed a substantially increased LC3-II in IF1 KO compared with WT hearts subjected to TAC (Fig. 6C). Protein levels of p62 were decreased and Beclin-1 were increased in IF1 KO compared to WT hearts after TAC (Fig. 6D,E). As a result, the ratio of p62 to Beclin-1 was markedly decreased (Fig. 6F). These results suggest that autophagic flux is activated in IF1 deficient hearts under the pressure overload condition.

## Discussion

We demonstrate for the first time that cardiac expression of IF1 is upregulated in pressure overload hearts. While there is no basal phenotype, the loss of IF1 protects the mouse heart from pressure overload hypertrophy. The loss of IF1 prevents excessive depolarization of the mitochondrial membrane and optimizes cardiac energy homeostasis with AMPK activation. These findings suggest a maladaptive response of IF1 upregulation in the chronically stressed heart.

It has been well documented that IF1 suppresses the hydrolysis activity of ATP synthase^8, 9, 11, 19^ under acidic conditions such as myocardial ischemia, thus preserving ATP. When mitochondrial depolarization occurs in pathological conditions, such as myocardial ischemia, cardiac IF1 is suggested to be activated to inhibit ATP hydrolysis of ATP synthase^8, 9^. Inhibiting ATP hydrolysis has been proposed to be a potential clinical strategy for treating various pathological conditions^15, 20^, including myocardial ischemia-reperfusion injury^13, 14^. IF1 mimetic compounds, e.g., BMS-199264 and BMS-250685, have been shown to enhance cardiac performance in isolated heart subjected to myocardial ischemia/reperfusion^13, 14, 21^. Before further exploiting IF1 as a therapeutic target for heart diseases, it is essential to obtain the *in vivo* expression and function of IF1 in the pathological heart. Since the nuclear factor (NF)-κB regulates the transcriptional expression of IF1^22^ and NF-κB has been well documented to be activated in the hypertrophied and failing hearts^23, 24^, the upregulation of IF1 is possibly derived from the activation of NF-κB. Protein kinase A (PKA)-mediated IF1 phosphorylation may reduce IF1’s binding to ATP synthase F1 sector^25^. It is plausible that IF1 KO may avoid a potentially harmful effect derived from the accumulation of unbound IF1 in the hypertrophic heart as a result of PKA activation. Further studies should clarify the upstream signaling pathways and how PKA phosphorylated IF1 may attribute to cardiac pathology.

The *in vivo* role of IF1 remains understudied. Recently, several mouse lines were generated with IF1 KO^17, 18^ or an inducible, neuron-specific overexpression of a mutant IF1 (H49K) with a higher binding affinity for the β-catalytic subunit of the ATP synthase^15^. Two independent IF1 KO lines similarly showed no basal phenotypic changes at least before aging^17, 18^, confirming that IF1 is not essential for normal development and reproduction of mice. It is interesting that neuron-specific overexpression of a mutant IF1 (H49K) with a higher binding affinity for the β-catalytic subunit of the ATP synthase^15^ is neuron-protective due to a preconditioning effect in response to elevated reactive oxygen species (ROS). Studies on cultured mammalian cells and zebrafish showed that IF1 deficiency may impair heme synthesis as a result of mitochondrial pH elevation following the reversed proton flow^16^. In contrast, a recent study showed that inhibiting IF1 is beneficial in protecting severe mitochondrial respiratory chain dysfunction by reducing mitochondrial depolarization and apoptotic cell death in hepatic cells^17^. Results from the present study appear to support the protective role of IF1 knockout in the pathological heart. The present study on the mouse model of IF1 KO provides new insights into the potential effects of blocking IF1 in the *in vivo* heart under a mechanical stress condition and sheds new light on the mechanistic actions of IF1 in regulating mitochondrial function and cell survival signaling.

Mitochondrial ATP synthase is an enzyme catalyzing the final coupling step of oxidative energy production. Alterations at this crucial step of oxidative phosphorylation have major impacts on cellular energy supply and mitochondrial energetic homeostasis. This is especially the case in the energy-craving heart under pathological conditions. Under hypoxic conditions, the mitochondrial ATP synthase hydrolyzes ATP to replenish protons from the matrix into the intermembrane space to maintain mitochondrial membrane potential^10, 13, 26^. Therefore, ATP synthase is one of the key regulating points of ΔΨm. Severe membrane depolarization not only impairs mitochondrial respiration, but also leads to programmed cell death, as well as a net loss of functional myocardium. At least in the *in vitro* cell culture experiment, IF1 overexpression depolarizes the mitochondria as predicted due to a repression of ATP hydrolysis. On the other hand, we observed a lesser degree of mitochondria depolarization in IF1 KO mitochondria and in IF1 knockdown H9C2 cells, which is most likely due to the increase of reversed proton flow into the mitochondrial intermembrane space as a consequence of elevated ATP hydrolysis. As a result, ΔΨm in IF1 KO hearts is ultimately more resistant to pressure overload and isoproterenol-induced stresses that lead to the depolarization and the subsequent cell death. IF1 may play a role in facilitating mitochondrial cristae formation and EM assessment of ultrastructure of IF1 KO liver showed loss of cristae density^27^. However, our study showed that mitochondrial ultrastructure is normal in IF1 KO hearts under both basal and pressure overload condition. While further studies are warranted, it is plausible that factors other than IF1 may be sufficient to maintain mitochondrial integrity in the heart.

The downside to sustaining ΔΨm by repressing IF1 is the elevated consumption of ATP, which in turn activates the energy sensor complex, AMPK. AMPK activation has been shown to promote cellular autophagy by inhibiting the mammalian target of rapamycin (mTOR) signaling^28^. On the other hand, we did not detect changes in the activity of AKT (a serine/threonine kinase also known as protein kinase B [PKB]). AKT is a downstream protein in the phosphatidylinositol 3-kinase (PI3K) pathway, which is known to activate mTOR signaling. As expected, we did detect an increase of LC3-II and beclin-1, and a decrease of p62 in IF1 KO hearts subjected to pressure overload. In addition to the evidence of increased degradation of p62, the markedly decreased p62 to beclin-1 ratio supports an elevating autophagic flux^29^.

In conclusion, the myocardial protective effect of IF1 deficiency is not only related to its effects of maintaining ΔΨm, but also to the AMPK-mediated upregulation of autophagy flux. Potential interventions that could inhibit IF1 should be beneficial. Therefore, IF1 inhibition may serve as a potential therapeutic target in treating pathological cardiac hypertrophy and heart failure.

## Methods

### Transverse aortic constriction (TAC) procedure to induce left ventricular (LV) pressure overload hypertrophy

Pressure overload-induced hypertrophy was created by transverse aortic constriction (TAC) as described previously^30^. Briefly, three-month-old (male and female) IF1 KO and WT mice (C57/B6) were anesthetized and maintained by isoflurane inhalation. After a minimal chest opening, the transverse aorta was ligated by tying a 6–0 silk suture against a 26-gauge needle. Sham mice underwent the same procedure without ligation of the aorta. The above mice were sacrificed 4 weeks after TAC or sham operation. All experimental procedures were conducted in accordance with the *Guide for Care and Use of Laboratory Animals* and approved by the Institutional Animal Care and Use Committee of the University of Alabama at Birmingham.

### Isoproterenol infusion in mice

Three-month-old IF1 KO and WT mice (C57/B6) were infused with isoproterenol (ISO) (60 mg/kg/day) for 14 days. Alzet miniosmotic pumps (no. 2002; Alza Corp., Mountain View, California) containing either ISO or PBS were surgically inserted subcutaneously on the back under isoflurane anesthesia. All mice were sacrificed 2 weeks later.

### Cardiac mitochondrial Isolation

Mitochondria were isolated using the Mitochondrial Isolation Kit (Pierce) according to the manufacturer’s instructions^31^. Briefly, LV tissues from a single heart were washed and minced in 800 μl of Reagent A plus protease inhibitor cocktail (Sigma). The minced tissue was stroked 10 times with a Dounce homogenizer. The homogenate was centrifuged for 10 min at 700 g in 4 °C. The supernatant solution was transferred to a new chilled tube, and centrifuged for 15 min at 3,000 g in 4 °C. The cytosolic fraction was saved and stored at −80 °C and the pellet was washed with 500 μl of Wash Buffer and centrifuged for 5 min at 12,000 g in 4 °C. The final washed pellet was suspended in 100 μl of Reagent A containing protease inhibitor cocktail. Mitochondrial protein concentration was determined using the Lowry method.

### In-gel ATPase activity assay

BN-PAGE was used to resolve the native, intact mitochondrial protein complexes as described prevously^32^. ATP hydrolysis activity was measured following BN-PAGE as described above. After completion of BN-PAGE, the gel was briefly incubated in assay buffer (35 mmol/L Tris/Cl, 270 mmol/L glycine, 14 mmol/L MgSO_4_, 0.2% (w/v) Pb(NO_3_)_2_, 8 mmol/L ATP, pH 8.3) at varying time courses. Gels were fixed in 50% methanol and washed twice in ddH_2_O for 10 min and then scanned. The lead precipitates were quantified based on densitometry.

### Quantitative Real-time PCR

Total RNA samples were isolated from LV using a RNA extraction kit (Zymo) according to the manufacturer’s instructions. Quantitative real-time PCR analyses were carried out using the Step One Real-time PCR system (Applied Biosystems) to determine transcript levels of target genes. Expression of each gene was normalized to actin or 36B4, and compared across conditions. The primer pairs used for Real-Time PCR analyses were: IF1: TTCGGTGTCGGGGTATGAAG/GCCCGTATCCATGCTATCCG; Nppa: GGGGTAGGATTGACAGGAT/CGTGATAGATGAAGGCAGGAA; Nppb: GGGAGAACACGGCATCAT/GCCATTTCCTCCGACTTT; b-actin: CCAGCCTTCCTTCTTGGGTATG/TGCTGGAAGGTGGACAGTGAG; 36B4: TGGAGACAAGGTGGGAGCC/CACAGACAATGCCAGGACGC

### Protein extraction and Western blot analysis

We extracted total protein, mitochondrial and cytosolic proteins from mouse LV or cells, separated by SDS-PAGE, and performed immunoblot analyses. Human protein lysate was obtained from dilated cardiomyopathy patients and healthy donors, as approved by the UAB Institutional Review Board for Human Use. Informed consent for use of samples was obtained from all participants and/or their legal guardians. Proteins were transferred onto PVDF membranes and immunoblot analyses were carried out using antibodies from commercial sources: IF1 (Santa Cruz, mAb); pAMPK (CST, mAb); AMPK (CST, mAb); pAKT (CST, mAb); AKT (CST, mAb); caspase-3 (CST pAb); LC3 (MBL, pAb); p62 (CST, pAb); Beclin-1 (ThermoFisher, pAb); ATP5B (Abcam, mAb); actin (Sigma, mAb); GAPDH (Santa Cruz, pAb), following the manufacturer’s instructions. The immunoblotting images were captured with Chemidoc MP System (Bio-Rad) by developing the membranes in Luminata Western Chemiluminescent HRP Substrates (Millipore).

### Echocardiography measurement

We assessed the mouse cardiac structure and cardiac function using a high-resolution echocardiograph system (Visualsonic VEVO 770 System) as previously described^30, 33, 34^. Briefly, mice were anaesthetized with isoflurane inhalation. Heart rate was maintained at ~400 BPM and body temperature was maintained at 37 °C by placing mice on a heating pad. With a 35 MHz probe on long-axis and short axis M-mode images, we measured the thickness of interventricular septum (IVS) and LV posterior wall (LVPW), as well as LV internal diameter (LVID) at diastolic and systolic states. We analyzed the above data using the Advanced Cardiovascular Software package from the manufacturer to obtain parameters such as EF%, FS% and calibrated LV mass.

### Pathological examinations

Hearts were perfused and fixed using standard methods for pathological examinations by light microscope and transmission electron microscopy (TEM) that was processed in the High-Resolution Imaging Facility of the University of Alabama at Birmingham. Heart sections were processed and stained with hematoxylin/eosin (H/E) or Masson’s Trichrome blue in the Comparative Pathology Laboratory of the University of Alabama at Birmingham.

### JC-1 assay for mitochondria potential (ΔΨm)

Mitochondria potential (ΔΨm) was determined using the JC-1 assay kit (Sigma) according to the manufacturer’s instruction. Cultured H9C2 cells or isolated mitochondria (10 μg) from IF1 KO and WT mice were treated with 5 μM JC-1 for 10 minutes. The relative fluorescence of the sample was measured in a multimode microplate reader (Infinite 200, Tecan) with 485/535 nm excitation and 535/95 nm emission.

### Apoptosis detection

Apoptotic cardiomyocytes in frozen heart section were identified with TUNEL assay using the Click-iT TUNEL Alexa Fluor Imaging Assay Kit (Life technologies). Caspase-3 cleavage was detected via Western Blot analyses of protein expression, and analyzed as expression of cleaved caspase-3 compared to actin.

### Generation of stable ATPIF1 knockdown H9c2 cell line

H9C2 cardiomyocytes (ATCC CRL-1446, rat embryonic heart myoblasts) were transfected with short hairpin RNA (shRNA) constructs against ATPIF1 in pRS (shATPIF1-pRS) vector (OriGene Technologies, Rockville, MD) using Lipofectamine 2000 reagent (Invitrogen, Carlsbad, CA), according to the manufacturer’s instructions. The ATPIF1 targeting sequences that we validated for the successful knockdown of ATPIF1 expression are GGCGCTGGCTCCATCCGAGAAGCTGGTGG (cat# TG709527B) and ACTCGTCGGAGAGCATGGATTCGGGCGCT (cat# TG709527C). Forty-eight hours after transfection, the cells were selected with puromycin (0.5 µg/mL) in the growth DMEM medium for 4 weeks to obtain a stable cell line. Surviving cells were examined for the level of ATPIF1 expression by Western blot and further expanded for functional analysis.

### ATP assay

Relative ATP levels were assessed with the ATPlite Luminescence Assay System (PerkinElmer), according to the manufacturer’s instructions. Cultured H9C2 cells were treated with cell lysis and substrate solution. The relative luminescence of the sample was measured in a multimode microplate reader (Infinite 200, Tecan).

### Statistical analyses

Data for 2-group comparisons were analyzed with the nonparametric two-tailed Student *t*-test; otherwise, data were analyzed by one-factor or mixed, 2-factor ANOVA and multiple comparison using the GraphPad Prism 6 software (GraphPad Software Inc.). Values of quantitative results were expressed as mean ± SEM. Differences between groups and treatments were regarded as significant at p < 0.05.

## Electronic supplementary material

Supplementary Information
