# Supplementary material for: Knockout of the ATPase inhibitory factor 1 protects the heart from pressure overload-induced cardiac hypertrophy
Source: Sci Rep. 2017 Sep 5;7:10501. doi: 10.1038/s41598-017-11251-8 (PMC5585346; doi:10.1038/s41598-017-11251-8)
Supplement: Supplementary file 1 — Supplementary Information [file 41598_2017_11251_MOESM1_ESM.pdf]

Knockout of the ATPase inhibitory factor 1 protects the heart from pressure overload-induced cardiac hypertrophy

Kevin Yang[1], Qinqiang Long[1,2], Kamalamma Saja[1,3], Fengyuan Huang[1], Steven M. Pogwizd[4], Lufang Zhou[4], Masasuke Yoshida[5], Qinglin Yang[1, 2#]

Short title: IF1 knockout represses cardiac hypertrophy

[1]Department of Nutrition Science, University of Alabama at Birmingham,

Birmingham, Alabama 35294, USA

[2]Departments of Internal Medicine and Institute of Hypertension, Tongji Hospital, Tongji Medical College, Huazhong University of Science and Technology, Wuhan, P.R. China

[3]Department of Biochemistry, University of Kerala, Thiruvananthapuram, Kerala – 695 581, India.

[4]Division of Cardiovascular Diseases, Department of Medicine, University of Alabama at Birmingham, Birmingham, Alabama 35294, USA

[5]Department of Molecular Bioscience, Kyoto Sangyo University, Kamigamo-Motoyama, Kyoto 603-8555, Japan

Qinglin Yang, MD, PhD

Professor of Nutrition Sciences

Department of Nutrition Sciences

University of Alabama at Birmingham

1675 University Blvd, Webb 435

Birmingham, AL 35294-3360

Phone: 205-996-6022

Fax: 205-934-7049

E-mail: qyang@uab.edu

Correspondence should be addressed to Q.Y. (qyang@uab.edu)

Supplementary Figure 1

A.

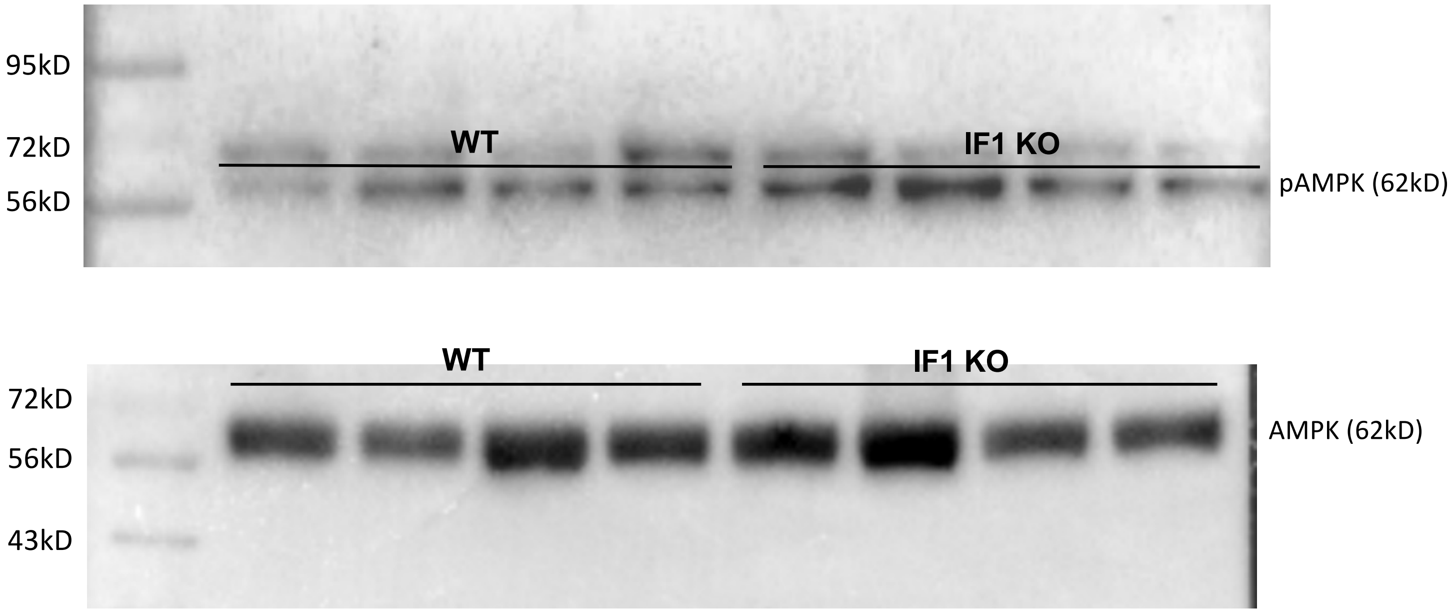

B.

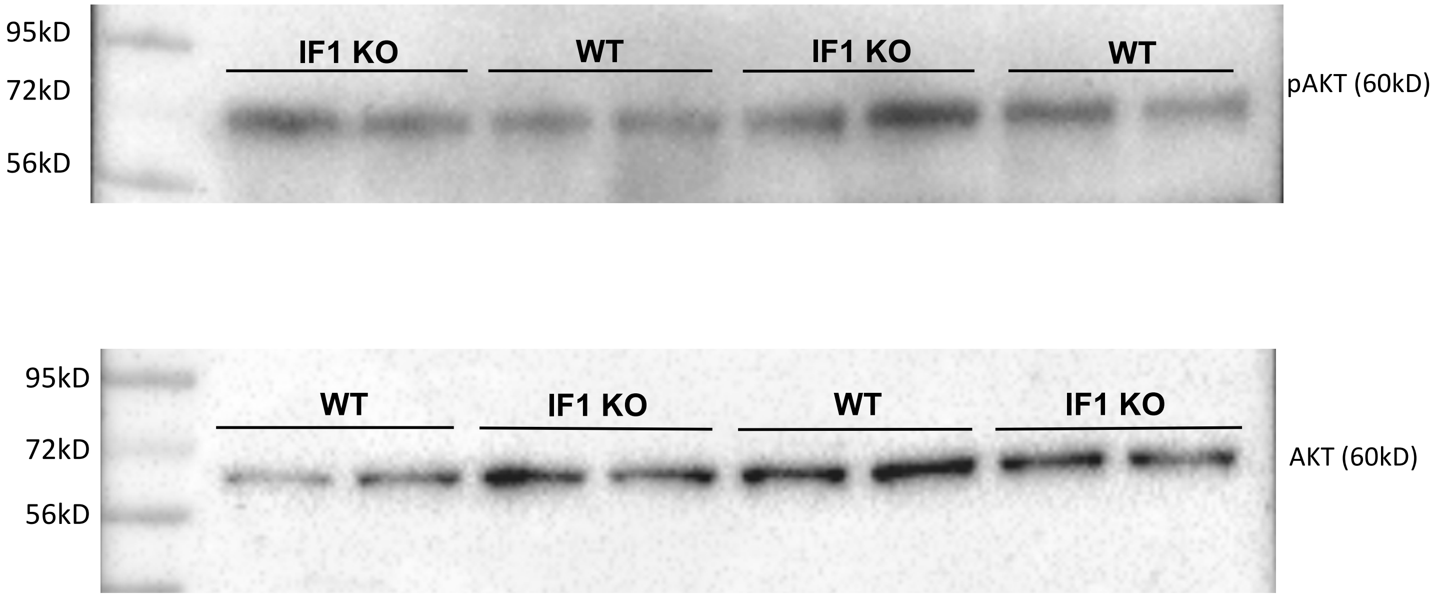

Supplementary Figure 1. Original blot of Figure 6A and 6B. Western blot images in the manuscript were cropped to exclude signals of prior blotting.

Supplementary Figure 2

A.

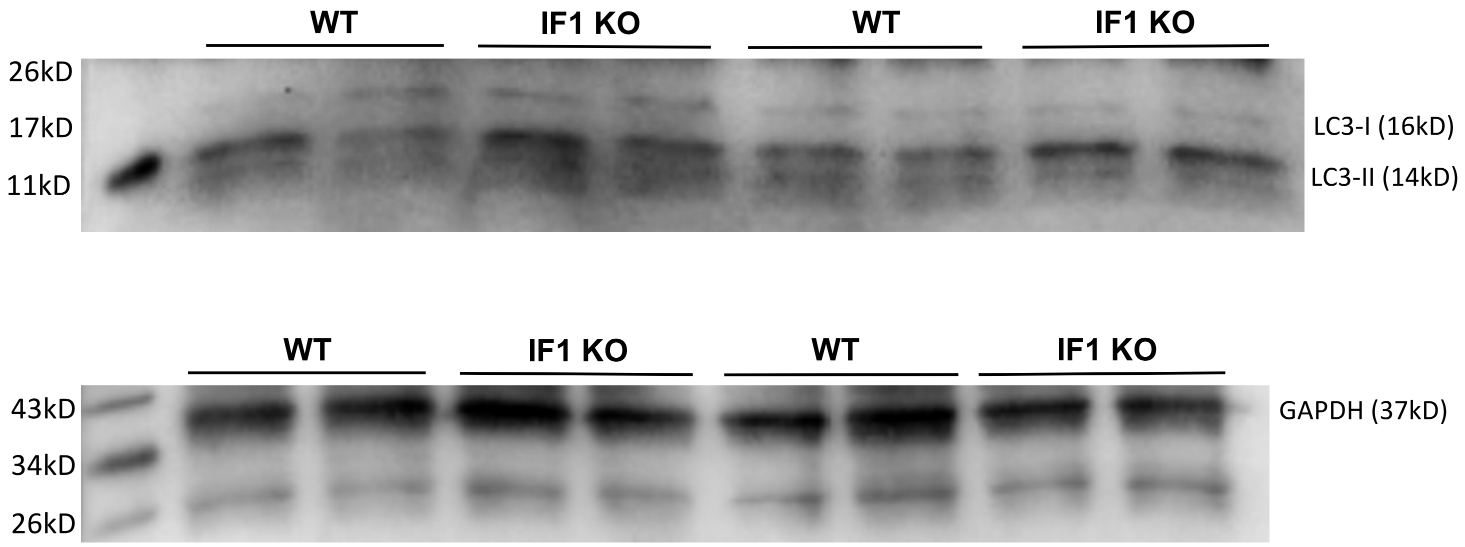

B.

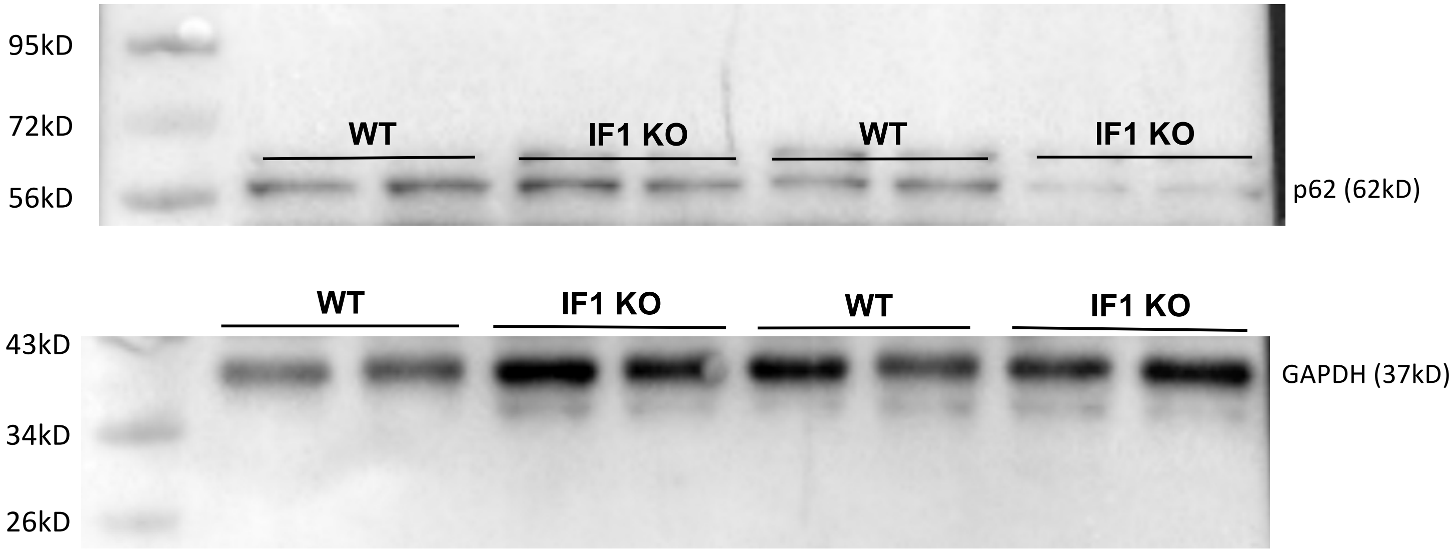

Supplementary Figure 2. Original blot of Figure 6C and 6D. Western blot images in the manuscript were cropped to exclude signals of prior blotting.

Supplementary Figure 3

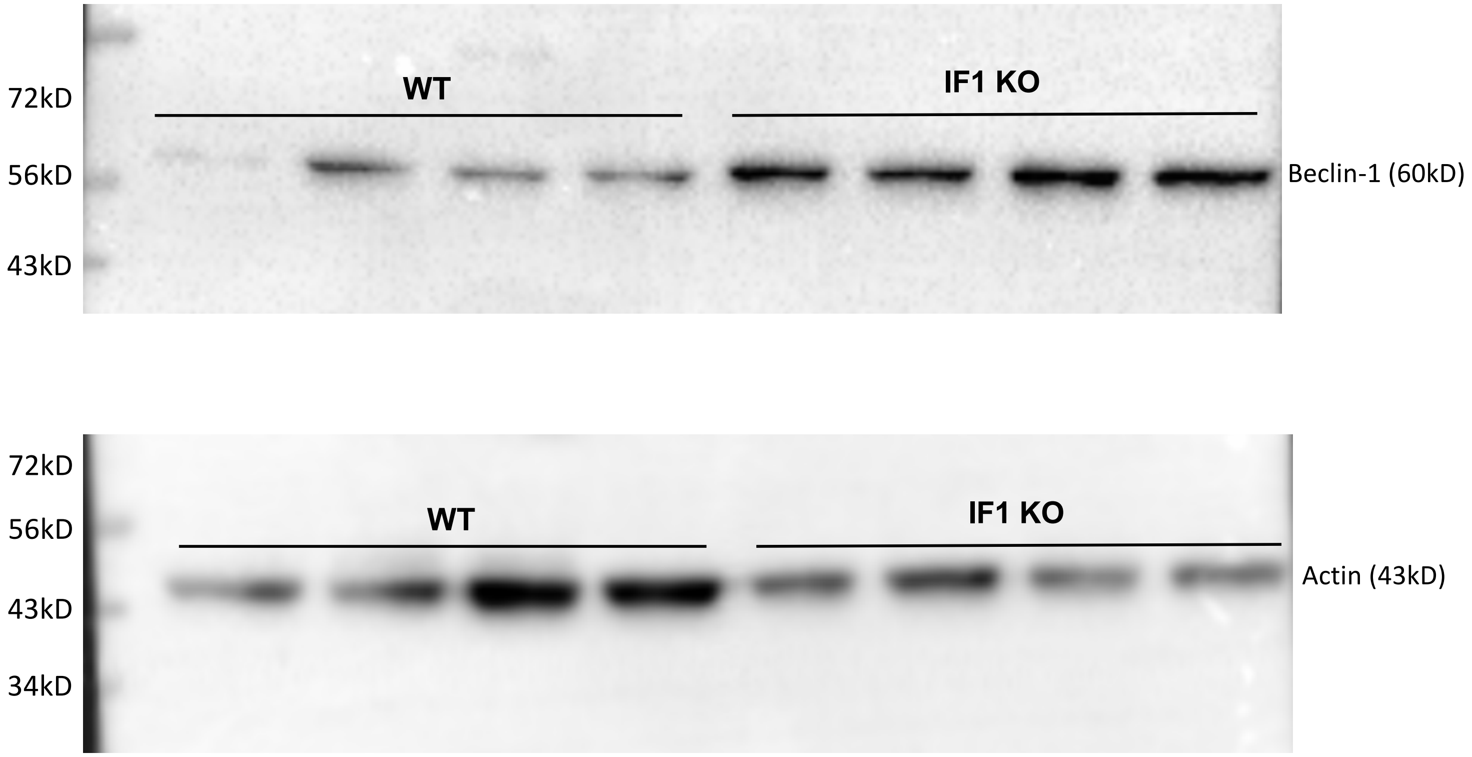

Supplementary Figure 3. Original blot of Figure 6E.
